# Entropy, Measures of Distance and Similarity of Q-Neutrosophic Soft Sets and Some Applications

**DOI:** 10.3390/e20090672

**Published:** 2018-09-05

**Authors:** Majdoleen Abu Qamar, Nasruddin Hassan

**Affiliations:** School of Mathematical Sciences, Faculty of Science and Technology, Universiti Kebangsaan Malaysia, Bangi, Selangor 43600, Malaysia

**Keywords:** decision making, distance measure, entropy measure, Q-neutrosophic soft set, similarity measure

## Abstract

The idea of the Q-neutrosophic soft set emerges from the neutrosophic soft set by upgrading the membership functions to a two-dimensional entity which indicate uncertainty, indeterminacy and falsity. Hence, it is able to deal with two-dimensional inconsistent, imprecise, and indeterminate information appearing in real life situations. In this study, the tools that measure the similarity, distance and the degree of fuzziness of Q-neutrosophic soft sets are presented. The definitions of distance, similarity and measures of entropy are introduced. Some formulas for Q-neutrosophic soft entropy were presented. The known Hamming, Euclidean and their normalized distances are generalized to make them well matched with the idea of Q-neutrosophic soft set. The distance measure is subsequently used to define the measure of similarity. Lastly, we expound three applications of the measures of Q-neutrosophic soft sets by applying entropy and the similarity measure to a medical diagnosis and decision making problems.

## 1. Introduction

The idea of fuzzy set theory established by Zadeh [1] is an important aspect in the study of uncertainty. The massive success of this theory has brought about the creation of many extensions of fuzzy sets such as the intuitionistic fuzzy set [2], interval-valued fuzzy set [3], vague set [4], and hesitant fuzzy set [5]. Smarandache [6,7] introduced a new model called neutrosophic set theory which refers to neutral knowledge. The innovative concept of neutrosophic set was presented to cater for indeterminate information which were conspicuously absent in the realm of fuzzy set and intuitionistic fuzzy set. Neutrosophic set (NS) is identified by three independent membership functions which describe the degree of truth (T), the degree of indeterminacy (I), and the degree of falsity (F), whose values are real standard or non-standard subset of unit interval ${]}^{-}0,1^{+}[$ where ${}^{-}0=0-\epsilon ,1^{+}=1+\epsilon$, ϵ is an infinitesimal number. The truth and falsity membership functions in a NS are analogous to the membership and nonmembership functions in an intuitionistic fuzzy set, and expresses the degree of belongingness and non-belongingness of the elements, whereas the indeterminacy membership function expresses the degree of neutrality in the information. The tri-membership structure of NSs enables it to handle uncertain, inconsistent and indeterminate data using truth, indeterminacy and falsity memberships. Indeterminacy membership function enables NSs to handle the neutrality aspects of the data, which cannot be handled by fuzzy sets and its extensions. The independency of the membership functions makes NSs more applicable than intuitionistic fuzzy set or other fuzzy-based models in which values of the membership and non-membership functions are dependent on one another.

The theory of soft set established by Molodtsov [8], is highly regarded as a general mathematical tool used to cope with uncertainties based on the theory of adequate parameterization. Since then, several researchers [9,10] have discussed more properties on soft set. Soft set has been extended to several different hybrid models. The extension was started by Maji et al. [11] who conceptualized fuzzy soft sets. Then, Maji [12] combined the ideas of neutrosophic set (NS) and soft set to present neutrosophic soft set (NSS). The NSS has the capacity to illustrate and characterize the attributes together comprehensively, while preserving all the features of NS.

However, all these models cannot handle two-dimensional incompatible, uncertain and indeterminate information. This motivated researchers to extend these models to be able to such situations such as Q-fuzzy soft set [13,14], Q-intuitionistic fuzzy soft set [15] and Q-neutrosophic soft set (Q-NSS) [16]. Q-NSS is an extended version of neutrosophic soft set depicted by three independent membership functions of two-dimensions to solve problems that appear in real life. It provided an adequate parametrization tool to deal with the aspects of two-dimensional imprecise, indeterminate and inconsistent data which appear in most real life problems, which serves the two-dimensionality and indeterminacy simultaneously.

Entropy and similarity measures are two basic concepts in fuzzy set theory. Similarity measure is an important tool for determining the degree of similarity between two objects. Majumdar and Samanta [17] started the research on the information measures of soft sets by introducing two types of distance-based similarity measures between soft sets and subsequently demonstrated the application of these measures in a medical diagnosis problem. Liu et al. [18] established a general form of similarity measure and entropy for fuzzy soft sets based on fuzzy equivalences. Jiang et al. [19] defined several distance measures between intuitionistic fuzzy soft sets and introduced an axiomatic definition of intuitionistic entropy for an intuitionistic fuzzy soft set. Broumi and Smarandache [20] studied some similarity measures of neutrosophic sets. Ye [21] initiated similarity measures based on the distances of interval neutrosophic sets. On the other hand, entropy was initially suggested to measure the level of fuzziness [22] of fuzzy set, followed by its axiomatic definition [23], and the entropy of interval-valued fuzzy set and intuitionistic fuzzy soft set [24]. Selvachandran et al. [25] discussed the intuitionistic entropy of generalized intuitionistic fuzzy soft set. Majumdar and Samanta [26] proposed the entropy of neutrosophic sets and then the entropy of NSS was defined [27]. Recently, many researchers are paying more attention to different information measures and their applications on various hybrid models of fuzzy soft set [28,29,30,31,32,33,34,35,36,37,38,39,40,41].

Following toward this path, we intend to study the information measures of Q-NSSs. The Q-NSS [16] is capable of resolving issues in two-dimensional uncertain, indeterminate and inconsistent environment. However, the literature has not indicate any studies on measures of the distance and degree of fuzziness of two-dimensional uncertain, indeterminate and incompatible information. Accordingly, we will rectify these deficiencies, by introducing the entropy, the distance measure and the similarity measure of Q-NSSs, as a continuation of the work on Q-NSSs done in [16]. The application of entropy measure of Q-NSSs will be illustrated in a decision making process, and the use of measure of similarity will be illustrated using two problems in medical diagnosis and decision making settings to show the significance of these measures in two-dimensional indeterminate real environment.

## 2. Preliminaries

We recapitulate the idea of soft set [8], neutrosophic set [6] and present an overview of the Q-NSS model [16].

**Definition** **1** ([8]). *A pair (F,E) is called a soft set over X, where F is a mapping F:E→P(X). In other words, the soft set is a parameterized family of subsets of the set X.*

Smarandache [6] introduced neutrosophic set as an extension of fuzzy set [1], in the following three definitions of neutrosophic set, subset and complement of neutrosophic set.

**Definition** **2** ([6]). *A neutrosophic set *Γ* on the universe X is defined as*

$$\Gamma =\{\langle x,(T_{\Gamma }\left(x\right),I_{\Gamma }\left(x\right),F_{\Gamma }\left(x\right))\rangle :x\in X\},$$

*where $T_{\Gamma }\left(x\right)$ is the truth membership function, $I_{\Gamma }\left(x\right)$ is the indeterminacy membership function and $F_{\Gamma }\left(x\right)$ is the falsity membership function. The functions $T_{\Gamma }\left(x\right),I_{\Gamma }\left(x\right)$ and $F_{\Gamma }\left(x\right)$ are real standard or non-standard subsets of ${]}^{-}0,1^{+}[$. That is $T_{\Gamma }\left(x\right),I_{\Gamma }\left(x\right),F_{\Gamma }\left(x\right):X\to {]}^{-}0,1^{+}[\;and$${}^{-}0\leq T_{\Gamma }\left(x\right)+I_{\Gamma }\left(x\right)+F_{\Gamma }\left(x\right)\leq 3^{+}.$*

**Definition** **3** ([42]). *Let *Γ* and *Ψ* be two neutrosophic sets, then we say that *Γ* is a subset of *Ψ* denoted by Γ⊆Ψ if and only if $T_{\Gamma }\left(x\right)\leq T_{\Psi }\left(x\right)$, $I_{\Gamma }\left(x\right)\geq I_{\Psi }\left(x\right)$ and $F_{\Gamma }\left(x\right)\geq F_{\Psi }\left(x\right)$ for all x∈X.*

**Definition** **4** ([42]). *The complement of a neutrosophic set *Γ* in the universe X is denoted by $\Gamma^{c}$, where*

$$\Gamma^{c}=\left\{\langle x,(F_{\Gamma }\left(x\right),I_{\Gamma }\left(x\right),T_{\Gamma }\left(x\right))\rangle :x\in X\right\}.$$

*The neutrosophic empty set $\Gamma_{0}$ in the universe X is* $\Gamma_{0}=\left\{\langle x,(0,1,1)\rangle :x\in X\right\}.$

Abu Qamar and Hassan [16] presented the idea of Q-neutrosophic set (Q-NS) to address two-dimensional inexact, indeterminate and incompatible data and extended this concept to multi Q-neutrosophic set and Q-neutrosophic soft set (Q-NSS).

**Definition** **5** **** ([16]). *Let X be a universal set and Q be a nonempty set. A Q-neutrosophic set $\Gamma_{Q}$ in X and Q is an object of the form*

$$\Gamma_{Q}=\left\{\langle \left(x,q\right),T_{\Gamma_{Q}}\left(x,q\right),I_{\Gamma_{Q}}\left(x,q\right),F_{\Gamma_{Q}}\left(x,q\right)\rangle :x\in X,q\in Q\right\},$$

*where $T_{\Gamma_{Q}},I_{\Gamma_{Q}},F_{\Gamma_{Q}}:X\times Q\to {]}^{-}0,1^{+}[$ are the true membership function, indeterminacy membership function and false membership function, respectively with ${}^{-}0\leq T_{\Gamma_{Q}}+I_{\Gamma_{Q}}+F_{\Gamma_{Q}}\leq 3^{+}$.*

**Definition** **6** ([16]). *Let X be a universal set, Q be any nonempty set, l be any positive integer and I be a unit interval [0,1]. A multi Q-neutrosophic set ${\tilde{\Gamma }}_{Q}$ in X and Q is a set of ordered sequences*

$${\tilde{\Gamma }}_{Q}=\left\{\langle \left(x,q\right),T_{{\tilde{\Gamma }}_{Q{}_{i}}}\left(x,q\right),I_{{\tilde{\Gamma }}_{Q{}_{i}}}\left(x,q\right),F_{{\tilde{\Gamma }}_{Q{}_{i}}}\left(x,q\right)\rangle :x\in X,q\in Q\;for\;all\;i=1,2,\ldots ,l\right\},$$

*where $T_{{\tilde{\Gamma }}_{Q{}_{i}}},I_{{\tilde{\Gamma }}_{Q{}_{i}}},F_{{\tilde{\Gamma }}_{Q{}_{i}}}:X\times Q\to I^{l}\;for\;all\;i=1,2,\ldots ,l$ are respectively, truth membership function, indeterminacy membership function and falsity membership function for each x∈Xandq∈Q and satisfy the condition*

$$0\leq T_{{\tilde{\Gamma }}_{Q{}_{i}}}+I_{{\tilde{\Gamma }}_{Q{}_{i}}}+F_{{\tilde{\Gamma }}_{Q{}_{i}}}\leq 3\;for\;all\;i=1,2,\ldots ,l$$

*where l is called the dimension of ${\tilde{\Gamma }}_{Q}$.*

**Definition** **7** ([16]). *Let X be a universal set, E be a set of parameters, and Q be a nonempty set. Let $\mu^{l}QNS\left(X\right)$ denote the set of all multi Q-neutrosophic sets on X with dimension l=1. Let A⊆E. A pair $\left(\Gamma_{Q},A\right)$ is called a Q-neutrosophic soft set (Q-NSS) over X, where $\Gamma_{Q}$ is a mapping given by*

$$\Gamma_{Q}:A\to \mu^{l}QNS\left(X\right)$$

*such that $\Gamma_{Q}\left(e\right)=\phi$ if e∉A. A Q-neutrosophic soft set (Q-NSS) can be represented by the set of ordered pairs*

$$\left(\Gamma_{Q},A\right)=\left\{\left(e,\Gamma_{Q}\left(e\right)\right):e\in A,\Gamma_{Q}\in \mu^{l}QNS\left(X\right)\right\}.$$

*The set of all Q-neutrosophic soft sets (Q-NSSs) in X and Q is denoted by QNSS(X).*

**Definition** **8** ([16]). *Let $\left(\Gamma_{Q},A\right),\left(\Psi_{Q},B\right)\in Q-NSS\left(X\right)$. Then $\left(\Psi_{Q},B\right)$ is a QNS subset of $\left(\Gamma_{Q},A\right)$, denoted by $\left(\Psi_{Q},B\right)\subseteq \left(\Gamma_{Q},A\right)$, if B⊆A and $\Psi_{Q}\left(x\right)\subseteq \Gamma_{Q}\left(x\right)$ for all x∈X.*

Henceforth, let $X=\{x_{1},x_{2},\ldots ,x_{m}\}$ becomes a universe of discourse, $Q=\{q_{1},q_{2},\ldots ,q_{l}\}$ be a nonempty set, $A=\{e_{1},e_{2},\ldots ,e_{n}\}$ is a set of parameters while $\left(\Gamma_{Q},A\right),\left(\Psi_{Q},A\right),\left(\Upsilon_{Q},A\right)\in Q-NSS\left(X\right)$ be the set of all Q-NSS in the universe *X* as defined below:

$$\begin{array}{cc} \left(\Gamma_{Q},A\right)=\{( & T_{\Gamma_{Q}\left(e_{j}\right)}{\left(x,q\right)}_{i},I_{\Gamma_{Q}\left(e_{j}\right)}{\left(x,q\right)}_{i},F_{\Gamma_{Q}\left(e_{j}\right)}{\left(x,q\right)}_{i}):\forall e_{j}\in A,{\left(x,q\right)}_{i}\in X\times Q\},\\ \left(\Psi_{Q},A\right)=\{( & T_{\Psi_{Q}\left(e_{j}\right)}{\left(x,q\right)}_{i},I_{\Psi_{Q}\left(e_{j}\right)}{\left(x,q\right)}_{i},F_{\Psi_{Q}\left(e_{j}\right)}{\left(x,q\right)}_{i}):\forall e_{j}\in A,{\left(x,q\right)}_{i}\in X\times Q\},\\ \left(\Upsilon_{Q},A\right)=\{( & T_{\Upsilon_{Q}\left(e_{j}\right)}{\left(x,q\right)}_{i},I_{\Upsilon_{Q}\left(e_{j}\right)}{\left(x,q\right)}_{i},F_{\Upsilon_{Q}\left(e_{j}\right)}{\left(x,q\right)}_{i}):\forall e_{j}\in A,{\left(x,q\right)}_{i}\in X\times Q\}. \end{array}$$

## 3. Entropy of Q-Neutrosophic Soft Sets

We will propose in this section, the entropy of Q-NSS which measures the degree of fuzziness of a Q-NSS. This idea can be concreted in the following conditions, required for a Q-neutrosophic entropy:

(i) Will be null when the set is a Q-intuitionistic fuzzy soft set.
(ii) Will be maximum if the set is completely Q-neutrosophic soft set.
(iii) The Q-neutrosophic entropy of a Q-NSS and its complement is equal.
(iv) If the degree of membership, indeterminacy membership and non-membership of each element decrease, the sum will do so as well, and therefore, this set becomes fuzzier, and therefore the entropy should increase.

In view of the conditions stated above, we propose the following axiomatic definition for the Q-neutrosophic entropy of a Q-NSS.

**Definition** **9.** *A function $\varepsilon :Q-NSS\left(X\right)\to R^{+}\cup \left\{0\right\}$ is an entropy on $\left(\Gamma_{Q},A\right)\in Q-NSS\left(X\right)$ if the following properties are satisfied:*

(ε1) *$\varepsilon (\left(\Gamma_{Q},A\right))=0$ if and only if $\left(\Gamma_{Q},A\right)$ is a Q-intuitionistic fuzzy soft set,*
(ε2) *$\varepsilon (\left(\Gamma_{Q},A\right))=lmn$ if and only if $T_{\Gamma_{Q}\left(e_{j}\right)}{\left(x,q\right)}_{i}=I_{\Gamma_{Q}\left(e_{j}\right)}{\left(x,q\right)}_{i}=F_{\Gamma_{Q}\left(e_{j}\right)}{\left(x,q\right)}_{i}=0$, i.e., $\left(\Gamma_{Q},A\right)$ is completely Q-neutrosophic soft set for all $e_{j}\in A,{\left(x,q\right)}_{i}\in X\times Q$,*
(ε3) *$\varepsilon \left(\left(\Gamma_{Q},A\right)\right)=\varepsilon \left({\left(\Gamma_{Q},A\right)}^{c}\right)$ for all $\left(\Gamma_{Q},A\right)\in Q-NSS\left(X\right)$,*
(ε4) *If $\left(\Gamma_{Q},A\right)⪯\left(\Psi_{Q},A\right)$ then $\varepsilon \left(\left(\Gamma_{Q},A\right)\right)\geq \varepsilon \left(\left(\Psi_{Q},A\right)\right)$, where $\left(\Gamma_{Q},A\right)⪯\left(\Psi_{Q},A\right)$ if and only if $T_{\Gamma_{Q}\left(e\right)}\left(x,q\right)\leq T_{\Psi_{Q}\left(e\right)}\left(x,q\right),I_{\Gamma_{Q}\left(e\right)}\left(x,q\right)\leq I_{\Psi_{Q}\left(e\right)}\left(x,q\right)$ and $F_{\Gamma_{Q}\left(e\right)}\left(x,q\right)\leq F_{\Psi_{Q}\left(e\right)}\left(x,q\right)$ for all e∈A and (x,q)∈X×Q.*

Now we prove that the entropy of a Q-NSS has its maximum value if the Q-NSS is completely Q-neutrosophic soft set.

**Theorem** **1.** *The entropy of $\left(\Gamma_{Q},A\right)$, $\varepsilon (\left(\Gamma_{Q},A\right))$ is maximum if and only if $\left(\Gamma_{Q},A\right)$ is completely Q-neutrosophic soft set.*

**Proof.**  Assume $\varepsilon (\left(\Gamma_{Q},A\right))$ be a maximum. Suppose for some $e_{j}\in A$ and ${\left(x,q\right)}_{i}\in X\times Q$ that $T_{\Gamma_{Q}\left(e_{j}\right)}{\left(x,q\right)}_{i}\neq 0$ or $I_{\Gamma_{Q}\left(e_{j}\right)}{\left(x,q\right)}_{i}\neq 0$ or $F_{\Gamma_{Q}\left(e_{j}\right)}{\left(x,q\right)}_{i}\neq 0$. Now, suppose that there exists a Q-NSS $\left(\Psi_{Q},A\right)$ with $T_{\Psi_{Q}\left(e_{j}\right)}{\left(x,q\right)}_{i}=\frac{1}{2}T_{\Gamma_{Q}\left(e_{j}\right)}{\left(x,q\right)}_{i},I_{\Psi_{Q}\left(e_{j}\right)}{\left(x,q\right)}_{i}=I_{\Gamma_{Q}\left(e_{j}\right)}{\left(x,q\right)}_{i}$ and $F_{\Psi_{Q}\left(e_{j}\right)}{\left(x,q\right)}_{i}=F_{\Gamma_{Q}\left(e_{j}\right)}{\left(x,q\right)}_{i}$. Then, $T_{\Psi_{Q}\left(e_{j}\right)}{\left(x,q\right)}_{i}\leq T_{\Gamma_{Q}\left(e_{j}\right)}{\left(x,q\right)}_{i},I_{\Psi_{Q}\left(e_{j}\right)}{\left(x,q\right)}_{i}\leq I_{\Gamma_{Q}\left(e_{j}\right)}{\left(x,q\right)}_{i},F_{\Psi_{Q}\left(e_{j}\right)}{\left(x,q\right)}_{i}\leq F_{\Gamma_{Q}\left(e_{j}\right)}{\left(x,q\right)}_{i}$, which implies that $\left(\Psi_{Q},A\right)⪯\left(\Gamma_{Q},A\right)$. Hence, by property (ε4) of Definition 9, $\varepsilon \left(\left(\Psi_{Q},A\right)\right)\geq \varepsilon \left(\left(\Gamma_{Q},A\right)\right)$ which contradicts the first assumption that $\varepsilon (\left(\Gamma_{Q},A\right))$ maximum, implying that $T_{\Gamma_{Q}\left(e_{j}\right)}{\left(x,q\right)}_{i}=I_{\Gamma_{Q}\left(e_{j}\right)}{\left(x,q\right)}_{i}=F_{\Gamma_{Q}\left(e_{j}\right)}{\left(x,q\right)}_{i}=0$ for all $e_{j}\in A$ and ${\left(x,q\right)}_{i}\in X\times Q$.

Conversely, Let $\left(\Gamma_{Q},A\right)$ be a Q-NSS(X) with $T_{\Gamma_{Q}\left(e_{j}\right)}{\left(x,q\right)}_{i}=I_{\Gamma_{Q}\left(e_{j}\right)}{\left(x,q\right)}_{i}=F_{\Gamma_{Q}\left(e_{j}\right)}{\left(x,q\right)}_{i}=0$ and let $\left(\Psi_{Q},A\right)$ be any Q-NSS(X). Since $T_{\Psi_{Q}\left(e_{j}\right)}{\left(x,q\right)}_{i}\geq 0,I_{\Psi_{Q}\left(e_{j}\right)}{\left(x,q\right)}_{i}\geq 0$ and $F_{\Psi_{Q}\left(e_{j}\right)}{\left(x,q\right)}_{i}\geq 0$ for all $e_{j}\in A,{\left(x,q\right)}_{i}\in X\times Q$ it is clear that $\left(\Gamma_{Q},A\right)⪯\left(\Psi_{Q},A\right)$. Hence, by property (ε4) of Definition 9, we have $\varepsilon \left(\left(\Gamma_{Q},A\right)\right)\geq \varepsilon \left(\left(\Psi_{Q},A\right)\right)$ which implies $\varepsilon (\left(\Gamma_{Q},A\right))$ is a maximum. Thus completing the proof. ☐

Next, we give definition of an expression which allows us to create entropies of Q-NSS(X).

**Definition** **10.** *Let M={(α,β,γ)∈[0,1]×[0,1]×[0,1]:α+β+γ≤1}. Assume Φ:M→[0,1] is a mapping, which satisfies the following conditions:*

*1.*  *Φ(α,β,γ)=1 if and only if α+β+γ=1,*
*2.*  *Φ(α,β,γ)=0 if and only if α=β=γ=0,*
*3.*  *Φ(α,β,γ)=Φ(γ,β,α),*
*4.*  *If $\alpha_{1}\leq \alpha_{2},\beta_{1}\leq \beta_{2}$ and $\gamma_{1}\leq \gamma_{2}$, then $\Phi \left(\alpha_{1},\beta_{1},\gamma_{1}\right)\leq \Phi \left(\alpha_{2},\beta_{2},\gamma_{2}\right)$.*

Examples of Φ functions which verify the previous conditions are:

1. Φ(α,β,γ)=α+β+γ,
2. $\Phi \left(\alpha ,\beta ,\gamma \right)={\left(\alpha +\beta +\gamma \right)}^{k},k=2,3,\ldots$,

The following theorem gives an expression which will allow us to construct different Q-neutrosophic entropies using a mapping satisfies the conditions of Definition 10.

**Theorem** **2.** *Let $\left(\Gamma_{Q},A\right)$ be a Q-NSS, $\varepsilon :Q-NSS\left(X\right)\to R^{+}\cup \left\{0\right\}$ be a mapping and *Φ* be a mapping satisfying given condition in Definition 10. Then*

$$\varepsilon \left(\Gamma_{Q},A\right)=\sum_{j=1}^{n}\sum_{i=1}^{lm}\left(1-\Phi \left(T_{\Gamma_{Q}\left(e_{j}\right)}{\left(x,q\right)}_{i},I_{\Gamma_{Q}\left(e_{j}\right)}{\left(x,q\right)}_{i},F_{\Gamma_{Q}\left(e_{j}\right)}{\left(x,q\right)}_{i}\right)\right)$$

*is a Q-neutrosophic entropy for $\left(\Gamma_{Q},A\right)$.*

**Proof.**  We shall prove all conditions of Definition 9.

1. Let $\varepsilon (\Gamma_{Q},A)=0$. Then $\sum_{j=1}^{n}\sum_{i=1}^{lm}\left(1-\Phi \left(T_{\Gamma_{Q}\left(e_{j}\right)}{\left(x,q\right)}_{i},I_{\Gamma_{Q}\left(e_{j}\right)}{\left(x,q\right)}_{i},F_{\Gamma_{Q}\left(e_{j}\right)}{\left(x,q\right)}_{i}\right)\right)=0$ if and only if $\Phi (T_{\Gamma_{Q}\left(e_{j}\right)}{\left(x,q\right)}_{i},I_{\Gamma_{Q}\left(e_{j}\right)}{\left(x,q\right)}_{i},F_{\Gamma_{Q}\left(e_{j}\right)}{\left(x,q\right)}_{i}))=1$ for all $e_{j}\in A$ and ${\left(x,q\right)}_{i}\in X\times Q$ if and only if $T_{\Gamma_{Q}\left(e_{j}\right)}{\left(x,q\right)}_{i}+I_{\Gamma_{Q}\left(e_{j}\right)}{\left(x,q\right)}_{i}+F_{\Gamma_{Q}\left(e_{j}\right)}{\left(x,q\right)}_{i}=1$ for all $e_{j}\in A$ and ${\left(x,q\right)}_{i}\in X\times Q$. Then $\left(\Gamma_{Q},A\right)$ is a Q-intuitionistic fuzzy soft set.
2. Let $\varepsilon (\Gamma_{Q},A)=lmn$. Then $\sum_{j=1}^{n}\sum_{i=1}^{lm}\left(1-\Phi \left(T_{\Gamma_{Q}\left(e_{j}\right)}{\left(x,q\right)}_{i},I_{\Gamma_{Q}\left(e_{j}\right)}{\left(x,q\right)}_{i},F_{\Gamma_{Q}\left(e_{j}\right)}{\left(x,q\right)}_{i}\right)\right)=lmn$ if and only if $\Phi (T_{\Gamma_{Q}\left(e_{j}\right)}{\left(x,q\right)}_{i},I_{\Gamma_{Q}\left(e_{j}\right)}{\left(x,q\right)}_{i},F_{\Gamma_{Q}\left(e_{j}\right)}{\left(x,q\right)}_{i})$=0 for all $e_{j}\in A$ and ${\left(x,q\right)}_{i}\in X\times Q$ if and only if $T_{\Gamma_{Q}\left(e_{j}\right)}{\left(x,q\right)}_{i}=I_{\Gamma_{Q}\left(e_{j}\right)}{\left(x,q\right)}_{i}=F_{\Gamma_{Q}\left(e_{j}\right)}{\left(x,q\right)}_{i}=0.$
3. Let $\left(\Gamma_{Q},A\right)=\left\{\langle e_{j},\left(T_{\Gamma_{Q}\left(e\right)}{\left(x,q\right)}_{i},I_{\Gamma_{Q}\left(e_{j}\right)}{\left(x,q\right)}_{i},F_{\Gamma_{Q}\left(e_{j}\right)}{\left(x,q\right)}_{i}\right)\rangle :e_{j}\in A,{\left(x,q\right)}_{i}\in X\times Q\right\}$. Then
  $${\left(\Gamma_{Q},A\right)}^{c}=\left\{\langle e_{j},\left(F_{\Gamma_{Q}\left(e_{j}\right)}{\left(x,q\right)}_{i},I_{\Gamma_{Q}\left(e_{j}\right)}{\left(x,q\right)}_{i},T_{\Gamma_{Q}\left(e\right)}{\left(x,q\right)}_{i}\right)\rangle :e_{j}\in A,{\left(x,q\right)}_{i}\in X\times Q\right\}.$$
  Hence,
  $$\begin{array}{cc} \varepsilon \left(\Gamma_{Q},A\right)=\sum_{j=1}^{n}\sum_{i=1}^{lm}( & 1-\Phi (T_{\Gamma_{Q}\left(e_{j}\right)}{\left(x,q\right)}_{i},I_{\Gamma_{Q}\left(e_{j}\right)}{\left(x,q\right)}_{i},F_{\Gamma_{Q}\left(e_{j}\right)}{\left(x,q\right)}_{i}))\\ =\sum_{j=1}^{n}\sum_{i=1}^{lm}( & 1-\Phi (F_{\Gamma_{Q}\left(e_{j}\right)}{\left(x,q\right)}_{i},I_{\Gamma_{Q}\left(e_{j}\right)}{\left(x,q\right)}_{i},T_{\Gamma_{Q}\left(e\right)}{\left(x,q\right)}_{i}))\\ =\varepsilon (\Gamma_{Q},A & {)}^{c}. \end{array}$$
4. If $\left(\Gamma_{Q},A\right)⪯\left(\Psi_{Q},A\right)$, then it is clear that for all $e_{j}\in A$ and ${\left(x,q\right)}_{i}\in X\times Q$,
  $$\begin{array}{cc} T_{\Gamma_{Q}\left(e_{j}\right)}{\left(x,q\right)}_{i} & \leq T_{\Psi_{Q}\left(e_{j}\right)}{\left(x,q\right)}_{i},\\ I_{\Gamma_{Q}\left(e_{j}\right)}{\left(x,q\right)}_{i} & \leq I_{\Psi_{Q}\left(e_{j}\right)}{\left(x,q\right)}_{i}\;\mathrm{and}\\ F_{\Gamma_{Q}\left(e_{j}\right)}{\left(x,q\right)}_{i} & \leq F_{\Psi_{Q}\left(e_{j}\right)}{\left(x,q\right)}_{i}. \end{array}$$

Hence, we have

$$\Phi \left(T_{\Gamma_{Q}\left(e_{j}\right)}{\left(x,q\right)}_{i},I_{\Gamma_{Q}\left(e_{j}\right)}{\left(x,q\right)}_{i},F_{\Gamma_{Q}\left(e_{j}\right)}{\left(x,q\right)}_{i}\right)\leq \Phi \left(T_{\Psi_{Q}\left(e_{j}\right)}{\left(x,q\right)}_{i},I_{\Psi_{Q}\left(e_{j}\right)}{\left(x,q\right)}_{i},F_{\Psi_{Q}\left(e_{j}\right)}{\left(x,q\right)}_{i}\right)$$

and so

$$1-\Phi \left(T_{\Psi_{Q}\left(e_{j}\right)}{\left(x,q\right)}_{i},I_{\Psi_{Q}\left(e_{j}\right)}{\left(x,q\right)}_{i},F_{\Psi_{Q}\left(e_{j}\right)}{\left(x,q\right)}_{i}\right)\leq 1-\Phi \left(T_{\Gamma_{Q}\left(e_{j}\right)}{\left(x,q\right)}_{i},I_{\Gamma_{Q}\left(e_{j}\right)}{\left(x,q\right)}_{i},F_{\Gamma_{Q}\left(e_{j}\right)}{\left(x,q\right)}_{i}\right)$$

for all $e_{j}\in A$ and ${\left(x,q\right)}_{i}\in X\times Q.$ Thus, $\sum_{j=1}^{n}\sum_{i=1}^{lm}1-\Phi \left(T_{\Psi_{Q}\left(e_{j}\right)}{\left(x,q\right)}_{i},I_{\Psi_{Q}\left(e_{j}\right)}{\left(x,q\right)}_{i},F_{\Psi_{Q}\left(e_{j}\right)}{\left(x,q\right)}_{i}\right)\leq \sum_{j=1}^{n}\sum_{i=1}^{lm}1-\Phi (T_{\Gamma_{Q}\left(e_{j}\right)}{\left(x,q\right)}_{i},$ $I_{\Gamma_{Q}\left(e_{j}\right)}{\left(x,q\right)}_{i},F_{\Gamma_{Q}\left(e_{j}\right)}{\left(x,q\right)}_{i})$, that is, $\varepsilon \left(\Gamma_{Q},A\right)\geq \varepsilon \left(\Psi_{Q},A\right)$. ☐

In the following, we give some examples of Q-neutrosophic entropy for Q-NSS.

(1) $$\begin{array}{cc} \varepsilon_{1}\left(\Gamma_{Q},A\right)= & \sum_{j=1}^{n}\sum_{i=1}^{lm}\left(1-\left(T_{\Gamma_{Q}\left(e_{j}\right)}{\left(x,q\right)}_{i}+I_{\Gamma_{Q}\left(e_{j}\right)}{\left(x,q\right)}_{i}+F_{\Gamma_{Q}\left(e_{j}\right)}{\left(x,q\right)}_{i}\right)\right). \end{array}$$

(2) $$\begin{array}{cc} \varepsilon_{2}\left(\Gamma_{Q},A\right)= & \sum_{j=1}^{n}\sum_{i=1}^{lm}\left(1-{\left(T_{\Gamma_{Q}\left(e_{j}\right)}{\left(x,q\right)}_{i}+I_{\Gamma_{Q}\left(e_{j}\right)}{\left(x,q\right)}_{i}+F_{\Gamma_{Q}\left(e_{j}\right)}{\left(x,q\right)}_{i}\right)}^{k}\right),k=2,3,\ldots \end{array}$$

In the following, we shall compare the performance of the proposed entropy measures by the following example:

**Example** **1.** *Let $\left(\Gamma_{Q},A\right)=\left\{\langle e,T_{\Gamma_{Q}\left(e\right)}\left(x,q\right),I_{\Gamma_{Q}\left(e\right)}\left(x,q\right),F_{\Gamma_{Q}\left(e\right)}\left(x,q\right)\rangle :e\in A,\left(x,q\right)\in X\times Q\right\}$ be a Q-NSS in the finite universe $X=\{x_{1},x_{2},\ldots ,x_{m}\}$ with the nonempty set $Q=\{q_{1},q_{2},\ldots ,q_{l}\}$. Then we define the Q-NSS, ${\left(\Gamma_{Q},A\right)}^{n}$ for any positive real number, n, as follows:*

$${\left(\Gamma_{Q},A\right)}^{n}=\left\{\langle e,T_{\Gamma_{Q}\left(e\right)}^{n}\left(x,q\right),1-{\left(1-I_{\Gamma_{Q}\left(e\right)}\left(x,q\right)\right)}^{n},1-{\left(1-F_{\Gamma_{Q}\left(e\right)}\left(x,q\right)\right)}^{n}\rangle :e\in A,\left(x,q\right)\in X\times Q\right\}.$$

*When we consider the Q-NSS, $\left(\Gamma_{Q},A\right)$, in the finite universe $X=\{x_{1},x_{2}\}$ with $Q=\{q_{1},q_{2}\}$ and the set of parameters $A=\{e_{1},e_{2}\}$ as*

$$\begin{array}{cc} (\Gamma_{Q} & ,A)\\ =\{ & \langle e_{1},\left[\left(x_{1},q_{1}\right),0.4,0.1,0.1\right],\left[\left(x_{1},q_{2}\right),0.2,0.11,0.12\right],\left[\left(x_{2},q_{1}\right),0.2,0.1,0.12\right],\left[\left(x_{2},q_{2}\right),0.4,0.12,0.12\right]\rangle ,\\ e_{2}, & \left[\left(x_{1},q_{1}\right),0.1,0.15,0.1\right],\left[\left(x_{1},q_{2}\right),0.1,0.12,0.1\right],\left[\left(x_{2},q_{1}\right),0.3,0.15,0.13\right],\left[\left(x_{2},q_{2}\right),0.4,0.13,0.11\right]\}. \end{array}$$

*Using the above operation, we can generate the following Q-NSSs:*

$$\begin{array}{cc} (\Gamma_{Q} & {,A)}^{2}\\ =\{ & \langle e_{1},\left[\left(x_{1},q_{1}\right),0.16,0.19,0.19\right],\left[\left(x_{1},q_{2}\right),0.04,0.2079,0.2256\right],\left[\left(x_{2},q_{1}\right),0.04,0.19,0.2256\right],\\ & \left[\left(x_{2},q_{2}\right),0.16,0.2256,0.2256\right]\rangle ,e_{2},\left[\left(x_{1},q_{1}\right),0.01,0.2775,0.19\right],\left[\left(x_{1},q_{2}\right),0.01,0.2256,0.19\right],\\ & \left[\left(x_{2},q_{1}\right),0.09,0.2775,0.2431\right],\left[\left(x_{2},q_{2}\right),0.16,0.2431,0.2079\right]\}, \end{array}$$

$$\begin{array}{cc} (\Gamma_{Q} & {,A)}^{3}\\ =\{ & \langle e_{1},\left[\left(x_{1},q_{1}\right),0.064,0.271,0.271\right],\left[\left(x_{1},q_{2}\right),0.008,0.2950,0.3185\right],\left[\left(x_{2},q_{1}\right),0.008,0.271,0.3185\right],\\ & \left[\left(x_{2},q_{2}\right),0.064,0.3185,0.3185\right]\rangle ,e_{2},\left[\left(x_{1},q_{1}\right),0.001,0.3859,.271\right],\left[\left(x_{1},q_{2}\right),0.001,0.3185,0.271\right],\\ & \left[\left(x_{2},q_{1}\right),0.027,0.3859,0.3415\right],\left[\left(x_{2},q_{2}\right),0.064,0.3415,0.2950\right]\},\\ (\Gamma_{Q} & {,A)}^{4}\\ =\{ & \langle e_{1},\left[\left(x_{1},q_{1}\right),0.0256,0.3439,0.3439\right],\left[\left(x_{1},q_{2}\right),0.0016,0.3726,0.4003\right],\left[\left(x_{2},q_{1}\right),0.0016,0.3439,0.4003\right],\\ & \left[\left(x_{2},q_{2}\right),0.0256,0.4003,0.4003\right]\rangle ,e_{2},\left[\left(x_{1},q_{1}\right),0.0001,0.4780,0.3439\right],\left[\left(x_{1},q_{2}\right),0.0001,0.4003,0.3439\right],\\ & \left[\left(x_{2},q_{1}\right),0.0081,0.4780,0.4271\right],\left[\left(x_{2},q_{2}\right),0.0256,0.7271,0.3726\right]\}. \end{array}$$

*By taking into account the characterization of linguistic variable, $\left(\Gamma_{Q},A\right)$ may be viewed as “Large” in X×Q. Using the above operations, ${\left(\Gamma_{Q},A\right)}^{2}$ may be viewed as “Very Large”, ${\left(\Gamma_{Q},A\right)}^{3}$ may be viewed as “Quite Very Large” and ${\left(\Gamma_{Q},A\right)}^{4}$ may be viewed as “Very Very Large”. From logical consideration and human intuition, the proposed entropy measures shown in Equations (1) and (2) of these Q-NSSs is required to satisfy the ranking order $\varepsilon_{i}\left(\left(\Gamma_{Q},A\right)\right)>\varepsilon_{i}{\left(\Gamma_{Q},A\right)}^{n},i=1,2$.*

*Therefore, from calculated entropy values listed in Table 1, we can easily see that they satisfy the required ranking order.*

## 4. Measures of Distance and Similarity of Q-Neutrosophic Soft Sets

The axiomatic definitions of the distance measure and similarity measure will be introduced. We present several distance measures between Q-NSSs, specifically the distances of Hamming, Euclidean, normalized Hamming and normalized Euclidean. These distances are obtained through generalizing the corresponding measures of distances for NSSs characterized in [27], to make them appropriate to the basis of Q-NSSs.

We start with defining the axiomatic definition for the measure of distance between two Q-NSSs. Next the distances of Hamming, Euclidean, and their normalized forms are defined.

**Definition** **11.** *A real valued non-negative function $d:Q-NSS\left(X\right)\times Q-NSS\left(X\right)\to R^{+}\cup 0$ is a distance function between Q-NSSs if the conditions below are satisfied for any $\left(\Gamma_{Q},A\right),\left(\Psi_{Q},A\right),\left(\Upsilon_{Q},A\right)\in Q-NSSs\left(X\right)$*

*(d1)*  *$d(\left(\Gamma_{Q},A\right),\left(\Psi_{Q},A\right))\geq 0$,*
*(d2)*  *$d\left(\left(\Gamma_{Q},A\right),\left(\Psi_{Q},A\right)\right)=d\left(\left(\Psi_{Q},A\right),\left(\Gamma_{Q},A\right)\right)$,*
*(d3)*  *$d(\left(\Gamma_{Q},A\right),\left(\Psi_{Q},A\right))=0$ if and only if $\left(\Gamma_{Q},A\right)=\left(\Psi_{Q},A\right)$,*
*(d4)*  *$d\left(\left(\Gamma_{Q},A\right),\left(\Psi_{Q},A\right)\right)+d\left(\left(\Psi_{Q},A\right),\left(\Upsilon_{Q},A\right)\right)\geq d\left(\left(\Gamma_{Q},A\right),\left(\Upsilon_{Q},A\right)\right)$.*

**Definition** **12.** *If $X=\{x_{1},x_{2},\ldots ,x_{m}\}$ is a universal set, $Q=\{q_{1},q_{2},\ldots ,q_{l}\}$ is a nonempty set, $A=\{e_{1},e_{2},\ldots ,e_{n}\}$ being a set of parameters, $\left(\Gamma_{Q},A\right)$ and $\left(\Psi_{Q},A\right)$ are a Q-NSSs(X) and d a distance measure between Q-NSSs for all $e_{j}\in A$ and ${\left(x,q\right)}_{i}\in X\times Q$ which are given below:*

*(1)*  *The Hamming distance:*
  $$d_{Q-NSS}^{H}\left(\left(\Gamma_{Q},A\right),\left(\Psi_{Q},A\right)\right)=\sum_{j=1}^{n}\sum_{i=1}^{lm}\frac{|\Delta_{ij}T\left(x,q\right)|+|\Delta_{ij}I\left(x,q\right)|+|\Delta_{ij}F\left(x,q\right)|}{3},$$
  *where*
  $$\begin{array}{c} \Delta_{ij}T\left(x,q\right)=T_{\Gamma_{Q}\left(e_{j}\right)}{\left(x,q\right)}_{i}-T_{\Psi_{Q}\left(e_{j}\right)}{\left(x,q\right)}_{i},\\ \Delta_{ij}I\left(x,q\right)=I_{\Gamma_{Q}\left(e_{j}\right)}{\left(x,q\right)}_{i}-I_{\Psi_{Q}\left(e_{j}\right)}{\left(x,q\right)}_{i}\;\mathrm{and}\\ \Delta_{ij}F\left(x,q\right)=F_{\Gamma_{Q}\left(e_{j}\right)}{\left(x,q\right)}_{i}-F_{\Psi_{Q}\left(e_{j}\right)}{\left(x,q\right)}_{i}. \end{array}$$
*(2)*  *The normalized Hamming distance:*
  $$d_{Q-NSS}^{NH}\left(\left(\Gamma_{Q},A\right),\left(\Psi_{Q},A\right)\right)=\frac{d_{Q-NSS}^{H}\left(\left(\Gamma_{Q},A\right),\left(\Psi_{Q},A\right)\right)}{lmn}.$$
*(3)*  *The Euclidean distance:*
  $$d_{Q-NSS}^{E}\left(\left(\Gamma_{Q},A\right),\left(\Psi_{Q},A\right)\right)={\left(\sum_{j=1}^{n}\sum_{i=1}^{lm}\frac{{\left(\Delta_{ij}T\left(x,q\right)\right)}^{2}+{\left(\Delta_{ij}I\left(x,q\right)\right)}^{2}+{\left(\Delta_{ij}F\left(x,q\right)\right)}^{2}}{3}\right)}^{\frac{1}{2}}$$
  *where*
  $$\begin{array}{c} \Delta_{ij}T\left(x,q\right)=T_{\Gamma_{Q}\left(e_{j}\right)}{\left(x,q\right)}_{i}-T_{\Psi_{Q}\left(e_{j}\right)}{\left(x,q\right)}_{i},\\ \Delta_{ij}I\left(x,q\right)=I_{\Gamma_{Q}\left(e_{j}\right)}{\left(x,q\right)}_{i}-I_{\Psi_{Q}\left(e_{j}\right)}{\left(x,q\right)}_{i}\;and\\ \Delta_{ij}F\left(x,q\right)=F_{\Gamma_{Q}\left(e_{j}\right)}{\left(x,q\right)}_{i}-F_{\Psi_{Q}\left(e_{j}\right)}{\left(x,q\right)}_{i}. \end{array}$$
*(4)*  *The normalized Euclidean distance:*
  $$d_{Q-NSS}^{NE}\left(\left(\Gamma_{Q},A\right),\left(\Psi_{Q},A\right)\right)=\frac{d_{Q-NSS}^{E}\left(\left(\Gamma_{Q},A\right),\left(\Psi_{Q},A\right)\right)}{\sqrt{lmn}}.$$

The following properties clearly hold:

- $0\leq d_{Q-NSS}^{H}\left(\left(\Gamma_{Q},A\right),\left(\Psi_{Q},A\right)\right)\leq lmn.$
- $0\leq d_{Q-NSS}^{NH}\left(\left(\Gamma_{Q},A\right),\left(\Psi_{Q},A\right)\right)\leq 1.$
- $0\leq d_{Q-NSS}^{E}\left(\left(\Gamma_{Q},A\right),\left(\Psi_{Q},A\right)\right)\leq \sqrt{lmn}.$
- $0\leq d_{Q-NSS}^{NE}\left(\left(\Gamma_{Q},A\right),\left(\Psi_{Q},A\right)\right)\leq 1.$

Another main concept in the investigation of imprecise data is the measure of similarity. It shows the level of similarity between sets. Next, we define the similarity measure between Q-NSSs.

**Definition** **13.** *A real non-negative function S:Q−NSS(X)×Q−NSS(X)→[0,1] is a similarity measure between Q-NSSs if the following conditions are satisfied for any $\left(\Gamma_{Q},A\right),\left(\Psi_{Q},A\right),\left(\Upsilon_{Q},A\right)\in Q-NSS\left(X\right)$:*

*(S1)*  *$0\leq S(\left(\Gamma_{Q},A\right),\left(\Psi_{Q},A\right))\leq 1$,*
*(S2)*  *$S(\left(\Gamma_{Q},A\right),\left(\Psi_{Q},A\right))=1$ if and only if $\left(\Gamma_{Q},A\right)=\left(\Psi_{Q},A\right)$,*
*(S3)*  *$S\left(\left(\Gamma_{Q},A\right),\left(\Psi_{Q},A\right)\right)=S\left(\left(\Psi_{Q},A\right),\left(\Gamma_{Q},A\right)\right)$,*
*(S4)*  *If $\left(\Gamma_{Q},A\right)\subseteq \left(\Psi_{Q},A\right)\subseteq \left(\Upsilon_{Q},A\right)$, then $S\left(\left(\Gamma_{Q},A\right),\left(\Upsilon_{Q},A\right)\right)\leq \min\{S\left(\left(\Gamma_{Q},A\right),\left(\Psi_{Q},A\right)\right),$$S(\left(\Psi_{Q},A\right),\left(\Upsilon_{Q},A\right))\}.$*

In mathematics, the distance measures and similarity measures are connected ideas. Consequently, we can use the proposed distance measure to characterize similarity measures between Q-NSSs. Hence, we can characterize several similarity measures between Q-NSSs $\left(\Gamma_{Q},A\right)$ and $\left(\Psi_{Q},A\right)$ as follows:

(3) $$S_{Q-NSS}^{H}\left(\left(\Gamma_{Q},A\right),\left(\Psi_{Q},A\right)\right)=\frac{1}{1+d_{Q-NSS}^{H}\left(\left(\Gamma_{Q},A\right),\left(\Psi_{Q},A\right)\right)}$$

(4) $$S_{Q-NSS}^{E}\left(\left(\Gamma_{Q},A\right),\left(\Psi_{Q},A\right)\right)=\frac{1}{1+d_{Q-NSS}^{E}\left(\left(\Gamma_{Q},A\right),\left(\Psi_{Q},A\right)\right)}$$

(5) $$S_{Q-NSS}^{NH}\left(\left(\Gamma_{Q},A\right),\left(\Psi_{Q},A\right)\right)=\frac{1}{1+d_{Q-NSS}^{NH}\left(\left(\Gamma_{Q},A\right),\left(\Psi_{Q},A\right)\right)}$$

(6) $$S_{Q-NSS}^{NE}\left(\left(\Gamma_{Q},A\right),\left(\Psi_{Q},A\right)\right)=\frac{1}{1+d_{Q-NSS}^{NE}\left(\left(\Gamma_{Q},A\right),\left(\Psi_{Q},A\right)\right)}$$

The following example illustrates the proposed Hamming, Euclidean, normalized Hamming and normalized Euclidean distances between Q-NSSs and their corresponding similarity measures.

**Example** **2.** *Assume that two Q-NSS $\left(\Gamma_{Q},A\right)$ and $\left(\Psi_{Q},A\right)$ are defined as follows:*

$$\begin{array}{cc} \left(\Gamma_{Q},A\right)=\{\langle e_{1}, & \left[\left(x_{1},q_{1}\right),0.3,0.5,0.4\right],\left[\left(x_{1},q_{2}\right),0.1,0.2,0.3\right],\left[\left(x_{2},q_{1}\right),0.6,0.3,0.7\right],\left[\left(x_{2},q_{2}\right),0.8,0.1,0.3\right]\rangle ,\\ \langle e_{2}, & \left[\left(x_{1},q_{1}\right),0.8,0.2,0.5\right],\left[\left(x_{1},q_{2}\right),0.2,0.5,0.7\right],\left[\left(x_{2},q_{1}\right),0.4,0.1,0.9\right],\left[\left(x_{2},q_{2}\right),0.2,0.2,0.2\right]\rangle \},\\ \left(\Psi_{Q},A\right)=\{\langle e_{1}, & \left[\left(x_{1},q_{1}\right),0.1,0.6,0.5\right],\left[\left(x_{1},q_{2}\right),0.7,0.4,0.1\right],\left[\left(x_{2},q_{1}\right),0.3,0.1,0.1\right],\left[\left(x_{2},q_{2}\right),0.1,0.4,0.5\right]\rangle ,\\ \langle e_{2}, & \left[\left(x_{1},q_{1}\right),0.4,0.1,0.7\right],\left[\left(x_{1},q_{2}\right),0.2,0.6,0.2\right],\left[\left(x_{2},q_{1}\right),0.5,0.8,0.3\right],\left[\left(x_{2},q_{2}\right),0.9,0.4,0.1\right]\rangle \}. \end{array}$$

*By Definition 12 we have*

$$\begin{array}{cc} d_{Q-NSS}^{H}(\left(\Gamma_{Q},A\right),(\Psi_{Q},A & ))=\sum_{j=1}^{2}\sum_{i=1}^{4}\frac{|\Delta_{ij}T\left(x,q\right)|+|\Delta_{ij}I\left(x,q\right)|+|\Delta_{ij}F\left(x,q\right)|}{3}\\ & =\frac{\left|0.3-0.1|+|0.5-0.6|+|0.4-0.5\right|}{3}+\frac{\left|0.1-0.7|+|0.2-0.4|+|0.3-0.1\right|}{3}\\ & +\frac{\left|0.6-0.3|+|0.3-0.1|+|0.7-0.1\right|}{3}+\frac{\left|0.8-0.1|+|0.1-0.4|+|0.3-0.5\right|}{3}\\ & +\frac{\left|0.8-0.4|+|0.2-0.1|+|0.5-0.7\right|}{3}+\frac{\left|0.2-0.2|+|0.5-0.6|+|0.7-0.2\right|}{3}\\ & +\frac{\left|0.4-0.5|+|0.1-0.8|+|0.9-0.3\right|}{3}+\frac{\left|0.2-0.9|+|0.2-0.4|+|0.2-0.1\right|}{3}\\ & =2.3, \end{array}$$

*$d_{Q-NSS}^{NH}\left(\left(\Gamma_{Q},A\right),\left(\Psi_{Q},A\right)\right)=\frac{2.3}{2(2)(2)}=0.2875,$*

$$\begin{array}{cc} d_{Q-NSS}^{E}(\left(\Gamma_{Q},A\right) & ,\left(\Psi_{Q},A\right))={\left(\sum_{j=1}^{2}\sum_{i=1}^{4}\frac{{\left(\Delta_{ij}T\left(x,q\right)\right)}^{2}+{\left(\Delta_{ij}I\left(x,q\right)\right)}^{2}+{\left(\Delta_{ij}F\left(x,q\right)\right)}^{2}}{3}\right)}^{\frac{1}{2}}\\ & =(\frac{{\left(0.3-0.1\right)}^{2}+{\left(0.5-0.6\right)}^{2}+{\left(0.4-0.5\right)}^{2}}{3}+\frac{{\left(0.1-0.7\right)}^{2}+{\left(0.2-0.4\right)}^{2}+{\left(0.3-0.1\right)}^{2}}{3}\\ & +\frac{{\left(0.6-0.3\right)}^{2}+{\left(0.3-0.1\right)}^{2}+{\left(0.7-0.1\right)}^{2}}{3}+\frac{{\left(0.8-0.1\right)}^{2}+{\left(0.1-0.4\right)}^{2}+{\left(0.3-0.5\right)}^{2}}{3} \end{array}$$

$$\begin{array}{c} +\frac{{\left(0.8-0.4\right)}^{2}+{\left(0.2-0.1\right)}^{2}+{\left(0.5-0.7\right)}^{2}}{3}+\frac{{\left(0.2-0.2\right)}^{2}+{\left(0.5-0.6\right)}^{2}+{\left(0.7-0.2\right)}^{2}}{3}\\ +\frac{{\left(0.4-0.5\right)}^{2}+{\left(0.1-0.8\right)}^{2}+{\left(0.9-0.3\right)}^{2}}{3}+\frac{{\left(0.2-0.9\right)}^{2}+{\left(0.2-0.4\right)}^{2}+{\left(0.2-0.1\right)}^{2}}{3}{)}^{\frac{1}{2}}\\ =1.077 \end{array}$$

*and $d_{Q-NSS}^{NE}\left(\left(\Gamma_{Q},A\right),\left(\Psi_{Q},A\right)\right)=\frac{1.077}{\sqrt{2(2)(2)}}=0.380$. Now, by Equations (3)–(6), respectively, we have $S_{Q-NSS}^{H}\left(\left(\Gamma_{Q},A\right),\left(\Psi_{Q},A\right)\right)=\frac{1}{1+2.3}=0.303,$$S_{Q-NSS}^{NH}\left(\left(\Gamma_{Q},A\right),\left(\Psi_{Q},A\right)\right)=\frac{1}{1+0.2875}=0.777,$$S_{Q-NSS}^{E}\left(\left(\Gamma_{Q},A\right),\left(\Psi_{Q},A\right)\right)=\frac{1}{1+1.077}=0.481$ and $S_{Q-NSS}^{NE}\left(\left(\Gamma_{Q},A\right),\left(\Psi_{Q},A\right)\right)=\frac{1}{1+0.380}=0.725.$*

## 5. Applications

Q-NSSs is a suitable tool to better model and process imperfect two-dimensional information. In this section, we present a practical examples of Q-NSSs to show that the proposed entropy and similarity measures play a significant role in solving medical diagnosis and decision making problems. To facilitate the discussion, this section will be divided into two subsections. The first subsection will illustrate an application of the proposed entropy measure of Q-NSSs in decision making while the second subsection illustrates two applications of the proposed similarity measures of Q-NSSs in medical diagnosis and decision making.

### 5.1. Application of Entropy of Q-Neutrosophic Soft Sets

In the application of the proposed entropy measures in decision-making problems, if the entropy value for an alternative throughout all the attributes is smaller, the decision-maker can provide more useful information from this alternative. Hence, the alternative with the least entropy value should be assigned a significant preference and priority.

The following algorithm and subsequent example will illustrate the application of Q-neutrosophic soft entropy in decision making. Using the entropy defined in Theorem 2, we introduce the following approach to solve decision making problem followed by a real life example to show the validity of this approach.

**Step** **1:** Suppose there exists P alternatives represented by Q-NSSs.
**Step** **2:** Compute the entropy of $\left(\Gamma_{Q},A\right)$.
**Step** **3:** Select the smallest entropy such that the corresponding Q-NSS is the best choice.

**Example** **3.** *Suppose $X=\{x_{1},x_{2}\}$ is a set of houses, $Q=\{q_{1}=near\;the\;city,\;q_{2}=far\;from\;the\;city\}$ be a set which describe the place of a house, $A=\{e_{1}=\mathrm{ease}\;\mathrm{of}\;\mathrm{transport},\;e_{2}=\mathrm{large},\;e_{3}=\mathrm{beautiful}\}$ be a set of parameters. Suppose that Ms. L and Ms. M want to buy a house. The Q-NSSs $\left(\Gamma_{Q},A\right)$ and $\left(\Psi_{Q},A\right)$ describe the attractiveness of the house to them, respectively.*

$$\begin{array}{cc} \left(\Gamma_{Q},A\right)=\{\langle e_{1}, & \left[\left(x_{1},q_{1}\right),0.2,0.1,0.6\right],\left[\left(x_{1},q_{2}\right),0.5,0.4,0.1\right],\left[\left(x_{2},q_{1}\right),0.4,0.2,0.2\right],\left[\left(x_{2},q_{2}\right),0.2,0.2,0.2\right]\rangle ,\\ \langle e_{2}, & \left[\left(x_{1},q_{1}\right),0.1,0.5,0.2\right],\left[\left(x_{1},q_{2}\right),0.1,0.1,0.6\right],\left[\left(x_{2},q_{1}\right),0.3,0.3,0.3\right],\left[\left(x_{2},q_{2}\right),0.3,0.6,0.1\right]\rangle ,\\ \langle e_{3}, & \left[\left(x_{1},q_{1}\right),0.7,0.1,0.1\right],\left[\left(x_{1},q_{2}\right),0.3,0.2,0.3\right],\left[\left(x_{2},q_{1}\right),0.1,0.6,0.2\right],\left[\left(x_{2},q_{2}\right),0.2,0.7,0.1\right]\rangle \}, \end{array}$$

$$\begin{array}{cc} \left(\Psi_{Q},A\right)=\{\langle e_{1}, & \left[\left(x_{1},q_{1}\right),0.2,0.1,0.3\right],\left[\left(x_{1},q_{2}\right),0.4,0.3,0.2\right],\left[\left(x_{2},q_{1}\right),0.1,0.2,0.4\right],\left[\left(x_{2},q_{2}\right),0.1,0.4,0.4\right]\rangle ,\\ \langle e_{2}, & \left[\left(x_{1},q_{1}\right),0.2,0.2,0.5\right],\left[\left(x_{1},q_{2}\right),0.1,0.1,0.1\right],\left[\left(x_{2},q_{1}\right),0.4,0.3,0.2\right],\left[\left(x_{2},q_{2}\right),0.3,0.2,0.1\right]\rangle ,\\ \langle e_{3}, & \left[\left(x_{1},q_{1}\right),0.3,0.5,0.1\right],\left[\left(x_{1},q_{2}\right),0.1,0.1,0.4\right],\left[\left(x_{2},q_{1}\right),0.2,0.1,0.5\right],\left[\left(x_{2},q_{2}\right),0.3,0.1,0.3\right]\rangle \}. \end{array}$$

*As known, the less uncertainty information each attractiveness has, the larger possibility they will buy a house. Hence, we can rank buyers according to the entropy values of the corresponding Q-NSSs.*

*By Equation (1), we can compute $\varepsilon_{1}\left(\left(\Gamma_{Q},A\right)\right)=1.6$ and $\varepsilon_{1}\left(\left(\Psi_{Q},A\right)\right)=3.2$, i.e., $\varepsilon_{1}\left(\left(\Gamma_{Q},A\right)\right)<\varepsilon_{1}\left(\left(\Psi_{Q},A\right)\right)$. Therefore, from the computation above, the real estate agency can presume that Ms. L has a larger possibility to buy a house than Ms. M.*

### 5.2. Applications of Similarity Measure of Q-Neutrosophic Soft Sets

*We will now construct two algorithms to implement the defined Hamming similarity measure in medical diagnosis and decision making.*

#### 5.2.1. Similarity Measures of Q-Neutrosophic Soft Sets Applied to Medical Diagnosis

We will now show the implementation of the characterized Hamming similarity measure of Q-NSS in medical diagnosis.

The following algorithm to a medical diagnosis problem is used to determine if a patient suffers from cerebrum malignancy as we discuss in the next example.

**Step** **1:** Build a model Q-NSS for illness, which may be developed with the assistance of medicinal master person.
**Step** **2:** Construct Q-NSS for the patient.
**Step** **3:** Calculate distance between the model Q-NSS for illness and the Q-NSS for the patient.
**Step** **4:** Calculate similarity measure between the model Q-NSS for illness and the Q-NSS for the patient.
**Step** **5:** If the similarity measure is greater than or equal 0.5 then the patient may possibly suffer from the disease and if the similarity measure is less than 0.5 then the patient may not possibly suffer from the disease.

Below is a conceivable use of similarity measure of Q-NSS in a medical diagnosis to determine whether a patient having a few symptoms is experiencing cerebrum malignancy.

**Example** **4.** *Let $X=\{x_{1}=\mathrm{severe},\;x_{2}=\mathrm{mild}\}$ be a universal set which describes the intensity, $Q=\{q_{1}=\mathrm{frequently},\;q_{2}=\mathrm{rarely}\}$ describes the frequency of the symptoms. Let $A=\{e_{1}=\mathrm{headache},\;e_{2}=\mathrm{nausea},\;e_{3}=\mathrm{weakness}\}$ be a set of certain symptoms.*

***Step*** ***1:*** *Build the model Q-NSS for illness:*
  $$\begin{array}{cc} \left(\Pi_{Q},A\right)=\{\langle e_{1}, & \left[\left(x_{1},q_{1}\right),0.6,0.3,0.6\right],\left[\left(x_{1},q_{2}\right),0.8,0.1,0.5\right],\left[\left(x_{2},q_{1}\right),0.5,0.4,0.3\right],\left[\left(x_{2},q_{2}\right),0.3,0.2,0.2\right]\rangle ,\\ \langle e_{2}, & \left[\left(x_{1},q_{1}\right),0.7,0.2,0.4\right],\left[\left(x_{1},q_{2}\right),0.5,0.2,0.1\right],\left[\left(x_{2},q_{1}\right),0.3,0.2,0.1\right],\left[\left(x_{2},q_{2}\right),0.2,0.1,0.4\right]\rangle ,\\ \langle e_{3}, & \left[\left(x_{1},q_{1}\right),0.6,0.2,0.2\right],\left[\left(x_{1},q_{2}\right),0.7,0.4,0.1\right],\left[\left(x_{2},q_{1}\right),0.2,0.3,0.3\right],\left[\left(x_{2},q_{2}\right),0.4,0.3,0.2\right]\rangle \}. \end{array}$$
***Step*** ***2:*** *Construct Q-NSSs for patients Y and Z respectively as:*
  $$\begin{array}{cc} \left(\Gamma_{Q},A\right)=\{\langle e_{1}, & \left[\left(x_{1},q_{1}\right),0.8,0.3,0.6\right],\left[\left(x_{1},q_{2}\right),0.7,0.3,0.5\right],\left[\left(x_{2},q_{1}\right),0.4,0.2,0.1\right],\left[\left(x_{2},q_{2}\right),0.3,0.2,0.2\right]\rangle ,\\ \langle e_{2}, & \left[\left(x_{1},q_{1}\right),0.5,0.2,0.4\right],\left[\left(x_{1},q_{2}\right),0.5,0.3,0.1\right],\left[\left(x_{2},q_{1}\right),0.3,0.3,0.1\right],\left[\left(x_{2},q_{2}\right),0.2,0.2,0.3\right]\rangle ,\\ \langle e_{3}, & \left[\left(x_{1},q_{1}\right),0.6,0.2,0.5\right],\left[\left(x_{1},q_{2}\right),0.4,0.4,0.1\right],\left[\left(x_{2},q_{1}\right),0.2,0.1,0.2\right],\left[\left(x_{2},q_{2}\right),0.3,0.3,0.2\right]\rangle \}, \end{array}$$
  $$\begin{array}{cc} \left(\Psi_{Q},A\right)=\{\langle e_{1}, & \left[\left(x_{1},q_{1}\right),0.2,0.4,0.6\right],\left[\left(x_{1},q_{2}\right),0.1,0.4,0.3\right],\left[\left(x_{2},q_{1}\right),0.5,0.7,0.8\right],\left[\left(x_{2},q_{2}\right),0.6,0.5,0.6\right]\rangle ,\\ \langle e_{2}, & \left[\left(x_{1},q_{1}\right),0.1,0.2,0.4\right],\left[\left(x_{1},q_{2}\right),0.2,0.3,0.2\right],\left[\left(x_{2},q_{1}\right),0.6,0.5,0.8\right],\left[\left(x_{2},q_{2}\right),0.7,0.4,0.6\right]\rangle ,\\ \langle e_{3}, & \left[\left(x_{1},q_{1}\right),0.3,0.3,0.1\right],\left[\left(x_{1},q_{2}\right),0.2,0.1,0.4\right],\left[\left(x_{2},q_{1}\right),0.6,0.6,0.7\right],\left[\left(x_{2},q_{2}\right),0.8,0.5,0.7\right]\rangle \}. \end{array}$$
***Step*** ***3:*** *By Definition 12 the Hamming distance between $\left(\Pi_{Q},A\right)$ and $\left(\Gamma_{Q},A\right)$ is 0.867 while between $\left(\Pi_{Q},A\right)$ and $\left(\Psi_{Q},A\right)$ is 3.567.*
***Step*** ***4:*** *By Equation (3) the similarity measure between $\left(\Pi_{Q},A\right)$ and $\left(\Gamma_{Q},A\right)$ is 0.536 while between $\left(\Pi_{Q},A\right)$ and $\left(\Psi_{Q},A\right)$ is 0.219.*
***Step*** ***5:*** *Since $S(\left(\Pi_{Q},A\right),\left(\Gamma_{Q},A\right))>0.5$, therefore patient Y may possibly suffer cerebrum malignancy and since $S(\left(\Pi_{Q},A\right),\left(\Psi_{Q},A\right))<0.5$ therefore patient Z may not possibly suffer cerebrum malignancy.*

#### 5.2.2. Similarity Measures of Q-Neutrosophic Soft Sets Applied to Multicriteria Decision Making

We consider a method to show how to carry out the multicriteria decision-making problem by the defined similarity measure for Q-NSSs. Q-neutrosophic sets with a parametrization tool are favorable for decision making problems in the neutrosophic environment because of their ability to handle two-dimensional indeterminate problems. To solve a multicriteria decision-making problem which is based on the concept of the Q-neutrosophic soft set, we will consider the method introduced in [27] to show how to carry out a multicriteria decision making problem by the defined similarity measure for Q-NSSs. The two assessment criteria under consideration are the benefit and cost. To decide the best estimation of every criterion among every conceivable option, an ideal option can be distinguished by using a max-min-min operator and a min-max-max operator for the benefit and cost criteria, respectively. Then, for $\Gamma_{Q(e_{j})}{\left(x,q\right)}_{i}=\left(T_{\Gamma_{Q}\left(e_{j}\right)}{\left(x,q\right)}_{i},I_{\Gamma_{Q}\left(e_{j}\right)}{\left(x,q\right)}_{i},F_{\Gamma_{Q}\left(e_{j}\right)}{\left(x,q\right)}_{i}\right)$, we define an ideal Q-neutrosophic value for a benefit criterion and a cost criterion, respectively, as follows:

$$\begin{array}{cc} \Gamma_{Q}^{*}\left(e_{\mathrm{benefit}}\right){\left(x,q\right)}_{i}=\{( & T_{\Gamma_{Q}\left(e_{j}\right)}^{*}{\left(x,q\right)}_{i},I_{\Gamma_{Q}\left(e_{j}\right)}^{*}{\left(x,q\right)}_{i},F_{\Gamma_{Q}\left(e_{j}\right)}^{*}{\left(x,q\right)}_{i}):e_{j}\in A,{\left(x,q\right)}_{i}\in X\times Q\}\\ =\{( & \max_{j}T_{\Gamma_{Q}\left(e_{j}\right)}{\left(x,q\right)}_{i},\min_{j}I_{\Gamma_{Q}\left(e_{j}\right)}{\left(x,q\right)}_{i},\min_{j}F_{\Gamma_{Q}\left(e_{j}\right)}{\left(x,q\right)}_{i}):e_{j}\in A,{\left(x,q\right)}_{i}\in X\times Q\} \end{array}$$

*and*

$$\begin{array}{cc} \Gamma_{Q}^{*}\left(e_{\mathrm{cost}}\right){\left(x,q\right)}_{i}=\{( & T_{\Gamma_{Q}\left(e_{j}\right)}^{*}{\left(x,q\right)}_{i},I_{\Gamma_{Q}\left(e_{j}\right)}^{*}{\left(x,q\right)}_{i},F_{\Gamma_{Q}\left(e_{j}\right)}^{*}{\left(x,q\right)}_{i}):e_{j}\in A,{\left(x,q\right)}_{i}\in X\times Q\}\\ =\{( & \min_{j}T_{\Gamma_{Q}\left(e_{j}\right)}{\left(x,q\right)}_{i},\max_{j}I_{\Gamma_{Q}\left(e_{j}\right)}{\left(x,q\right)}_{i},\max_{j}F_{\Gamma_{Q}\left(e_{j}\right)}{\left(x,q\right)}_{i}):e_{j}\in A,{\left(x,q\right)}_{i}\in X\times Q\}. \end{array}$$

We can use the following algorithm to use the defined measures of similarity in decision making.

**Step** **1:** Construct Q-NSSs for the alternatives.
**Step** **2:** Construct the ideal Q-NSS from the Q-NSSs of the alternatives taking into account the benefit and the cost criteria.
**Step** **3:** Calculate the Hamming distance between the Q-NSS of the alternatives and the ideal Q-NSS.
**Step** **4:** Calculate the Hamming similarity measure between the Q-NSS of the alternatives and the ideal Q-NSS.
**Step** **5:** The decision is to select the Q-NSS which has the highest similarity to the ideal Q-NSS.

**Example** **5.** *Suppose that there is an investment company which has a downtrend in its returns. To overcome this situation, the administration need to put a rescue package into action. Four committees which are independent of one another, and an evaluation board are established by the administration. Each of these committees has prepared a contingency plan according to the following three criteria: $e_{1}$ is the hazard, $e_{2}$ is the development and $e_{3}$ is the ecological effect, where $e_{1}$ and $e_{3}$ are the cost criteria and $e_{2}$ is the benefit criterion. According to the reports offered separately by the evaluation board, the four possible alternatives are to be obtained under the above parameters by the form of Q-NSSs as follows:*

$$\begin{array}{cc} \left(\Gamma_{Q},A\right)=\{\langle e_{1}, & \left[\left(x_{1},q_{1}\right),0.5,0.2,0.4\right],\left[\left(x_{1},q_{2}\right),0.6,0.7,0.3\right],\left[\left(x_{2},q_{1}\right),0.2,0.5,0.5\right],\left[\left(x_{2},q_{2}\right),0.8,0.2,0.6\right]\rangle ,\\ \langle e_{2}, & \left[\left(x_{1},q_{1}\right),0.4,0.6,0.4\right],\left[\left(x_{1},q_{2}\right),0.4,0.5,0.5\right],\left[\left(x_{2},q_{1}\right),0.6,0.3,0.1\right],\left[\left(x_{2},q_{2}\right),0.4,0.1,0.1\right]\rangle ,\\ \langle e_{3}, & \left[\left(x_{1},q_{1}\right),0.8,0.3,0.6\right],\left[\left(x_{1},q_{2}\right),0.5,0.7,0.5\right],\left[\left(x_{2},q_{1}\right),0.9,0.8,0.1\right],\left[\left(x_{2},q_{2}\right),0.7,0.4,0.2\right]\rangle \}, \end{array}$$

$$\begin{array}{cc} \left(\Psi_{Q},A\right)=\{\langle e_{1}, & \left[\left(x_{1},q_{1}\right),0.6,0.6,0.6\right],\left[\left(x_{1},q_{2}\right),0.4,0.5,0.2\right],\left[\left(x_{2},q_{1}\right),0.2,0.3,0.9\right],\left[\left(x_{2},q_{2}\right),0.2,0.7,0.7\right]\rangle ,\\ \langle e_{2}, & \left[\left(x_{1},q_{1}\right),0.9,0.2,0.3\right],\left[\left(x_{1},q_{2}\right),0.1,0.1,0.4\right],\left[\left(x_{2},q_{1}\right),0.8,0.2,0.5\right],\left[\left(x_{2},q_{2}\right),0.6,0.6,0.3\right]\rangle ,\\ \langle e_{3}, & \left[\left(x_{1},q_{1}\right),0.7,0.1,0.5\right],\left[\left(x_{1},q_{2}\right),0.2,0.2,0.3\right],\left[\left(x_{2},q_{1}\right),0.2,0.8,0.4\right],\left[\left(x_{2},q_{2}\right),0.6,0.6,0.6\right]\rangle \}, \end{array}$$

$$\begin{array}{cc} \left(\Upsilon_{Q},A\right)=\{\langle e_{1}, & \left[\left(x_{1},q_{1}\right),0.3,0.4,0.9\right],\left[\left(x_{1},q_{2}\right),0.5,0.3,0.8\right],\left[\left(x_{2},q_{1}\right),0.6,0.2,0.2\right],\left[\left(x_{2},q_{2}\right),0.1,0.4,0.3\right]\rangle ,\\ \langle e_{2}, & \left[\left(x_{1},q_{1}\right),0.5,0.7,0.1\right],\left[\left(x_{1},q_{2}\right),0.9,0.2,0.2\right],\left[\left(x_{2},q_{1}\right),0.4,0.8,0.1\right],\left[\left(x_{2},q_{2}\right),0.5,0.9,0.3\right]\rangle ,\\ \langle e_{3}, & \left[\left(x_{1},q_{1}\right),0.9,0.4,0.4\right],\left[\left(x_{1},q_{2}\right),0.1,0.7,0.5\right],\left[\left(x_{2},q_{1}\right),0.5,0.4,0.1\right],\left[\left(x_{2},q_{2}\right),0.3,0.3,0.6\right]\rangle \} \end{array}$$

*and*

$$\begin{array}{cc} \left(\Lambda_{Q},A\right)=\{\langle e_{1}, & \left[\left(x_{1},q_{1}\right),0.7,0.1,0.1\right],\left[\left(x_{1},q_{2}\right),0.7,0.3,0.5\right],\left[\left(x_{2},q_{1}\right),0.3,0.4,0.1\right],\left[\left(x_{2},q_{2}\right),0.5,0.2,0.1\right]\rangle ,\\ \langle e_{2}, & \left[\left(x_{1},q_{1}\right),0.9,0.3,0.3\right],\left[\left(x_{1},q_{2}\right),0.3,0.4,0.3\right],\left[\left(x_{2},q_{1}\right),0.5,0.5,0.5\right],\left[\left(x_{2},q_{2}\right),0.7,0.2,0.4\right]\rangle ,\\ \langle e_{3}, & \left[\left(x_{1},q_{1}\right),0.8,0.6,0.3\right],\left[\left(x_{1},q_{2}\right),0.4,0.8,0.3\right],\left[\left(x_{2},q_{1}\right),0.2,0.7,0.9\right],\left[\left(x_{2},q_{2}\right),0.5,0.1,0.4\right]\rangle \}. \end{array}$$

*Using the approach created to acquire the most appropriate option, from the ideal Q-neutrosophic value, we can get the subsequent ideal Q-NSS:*

$$\begin{array}{cc} \left(\Pi_{Q},A\right)=\{\langle e_{1}, & \left[\left(x_{1},q_{1}\right),0.3,0.6,0.9\right],\left[\left(x_{1},q_{2}\right),0.4,0.7,0.8\right],\left[\left(x_{2},q_{1}\right),0.2,0.5,0.9\right],\left[\left(x_{2},q_{2}\right),0.1,0.7,0.7\right]\rangle ,\\ \langle e_{2}, & \left[\left(x_{1},q_{1}\right),0.9,0.2,0.1\right],\left[\left(x_{1},q_{2}\right),0.9,0.1,0.2\right],\left[\left(x_{2},q_{1}\right),0.8,0.2,0.1\right],\left[\left(x_{2},q_{2}\right),0.7,0.1,0.1\right]\rangle ,\\ \langle e_{3}, & \left[\left(x_{1},q_{1}\right),0.7,0.6,0.6\right],\left[\left(x_{1},q_{2}\right),0.1,0.8,0.5\right],\left[\left(x_{2},q_{1}\right),0.2,0.8,0.9\right],\left[\left(x_{2},q_{2}\right),0.3,0.6,0.6\right]\rangle \}. \end{array}$$

*Now, we can compute the similarity in view of the Hamming distance between the model ideal Q-NSS and the Q-NSS of each project as follows:*

$$\begin{array}{cc} d_{Q-NSS}^{H}\left(\left(\Gamma_{Q},A\right),\left(\Pi_{Q},A\right)\right)= & 3.3\Rightarrow S_{Q-NSS}^{H}\left(\left(\Gamma_{Q},A\right),\left(\Pi_{Q},A\right)\right)=0.233,\\ d_{Q-NSS}^{H}\left(\left(\Psi_{Q},A\right),\left(\Pi_{Q},A\right)\right)= & 2.133\Rightarrow S_{Q-NSS}^{H}\left(\left(\Psi_{Q},A\right),\left(\Pi_{Q},A\right)\right)=0.319,\\ d_{Q-NSS}^{H}\left(\left(\Upsilon_{Q},A\right),\left(\Pi_{Q},A\right)\right)= & 2.867\Rightarrow S_{Q-NSS}^{H}\left(\left(\Upsilon_{Q},A\right),\left(\Pi_{Q},A\right)\right)=0.259,\\ d_{Q-NSS}^{H}\left(\left(\Lambda_{Q},A\right),\left(\Pi_{Q},A\right)\right)= & 3.267\Rightarrow S_{Q-NSS}^{H}\left(\left(\Lambda_{Q},A\right),\left(\Pi_{Q},A\right)\right)=0.234. \end{array}$$

*Thus, the four alternatives are ranked orderly as $\left(\Psi_{Q},A\right)$, $\left(\Upsilon_{Q},A\right)$, $\left(\Lambda_{Q},A\right)$ and $\left(\Gamma_{Q},A\right)$. The administration should select the rescue with highest score, which is $\left(\Psi_{Q},A\right)$.*

## 6. Conclusions

We have introduced the entropy of Q-neutrosophic soft set which measures the degree of fuzziness of a Q-neutrosophic soft set, taking into account the two-dimensionality and indeterminacy of a data set, and have proposed several distance measures for Q-neutrosophic soft sets. Using the relations of distance measure to similarity measure, the corresponding similarity measures for Q-NSSs have been obtained. Finally, three different algorithms were constructed to implement the proposed measures in real life decision making issues to show the validity of these measures in a Q-neutrosophic soft environment. The advantages of the proposed measures are that they are defined over the set where the membership, indeterminacy membership and non-membership degrees are defined as a two-dimensional functions, and also complement the Q-NSS model in representing and modeling two-dimensional uncertain, indeterminate and inconsistent data. The measures proposed in this article may be extended further to different entropy, distance and similarity measures such as exponential entropy, cross entropy, Jaccard Measure, Cotangent Measure and others. Furthermore, the work presented in this paper can be used as a foundation to further extend the study of the information measures for structures obtained by incorporating the idea of Q-neutrosophic soft set to other concepts such as the refined neutrosophic set, soft expert set, bipolar/tripolar/multipolar neutrosophic, and many other structures.

## Figures and Tables

**Table 1:** Comparison of the fuzziness with different entropy measures.

${\left(\Gamma_{Q},A\right)}^{n}$	$\varepsilon_{1}$	$\varepsilon_{2},\;k=2$	$\varepsilon_{2},\;k=3$	$\varepsilon_{2},\;k=4$
$\left(\Gamma_{Q},A\right)$	4.02	5.8982	6.8354	7.3309
${\left(\Gamma_{Q},A\right)}^{2}$	3.795	5.7478	6.7720	7.3189
${\left(\Gamma_{Q},A\right)}^{3}$	2.771	4.5568	5.7158	6.4734
${\left(\Gamma_{Q},A\right)}^{4}$	1.6309	2.9011	3.8947	4.6756

## References

[1] Zadeh L.A. (1965). Fuzzy sets. Inf. Control.

[2] Atanassov K.T. (1986). Intuitionistic fuzzy sets. Fuzzy Sets Syst..

[3] Gorzalczany M.B. (1987). A method of inference in approximate reasoning based on interval-valued fuzzy sets. Fuzzy Sets Syst..

[4] Gau W.L., Buehrer D.J. (1993). Vague sets. IEEE Trans. Syst. Man Cybern..

[5] Torra V. (2010). Hesitant fuzzy sets. Int. J. Intell. Syst..

[6] Smarandache F. (2005). Neutrosophic set, a generalisation of the intuitionistic fuzzy sets. Int. J. Pure Appl. Math..

[7] Smarandache F. (1998). Neutrosophy: Neutrosophic Probability, Set, and Logic: Analytic Synthesis & Synthetic Analysis.

[8] Molodtsov D. (1999). Soft set theory-first results. Comput. Math. Appl..

[9] Danjuma S., Ismail M.A., Herawan T. (2017). An alternative approach to normal parameter reduction algorithm for soft set theory. IEEE Access.

[10] Ma X., Qin H. (2018). A distance-based parameter reduction algorithm of fuzzy soft sets. IEEE Access.

[11] Maji P.K., Biswas R., Roy A.R. (2001). Fuzzy soft sets. J. Fuzzy Math..

[12] Maji P.K. (2013). Neutrosophic soft set. Ann. Fuzzy Math. Inform..

[13] Adam F., Hassan N. (2014). Operations on Q-fuzzy soft set. Appl. Math. Sci..

[14] Adam F., Hassan N. (2014). Q-fuzzy soft set. Appl. Math. Sci..

[15] Broumi S. (2015). Q-intuitionistic fuzzy soft sets. J. New Theory.

[16] Abu Qamar M., Hassan N. (2018). Q-Neutrosophic soft relation and its application in decision making. Entropy.

[17] Majumdar P., Samanta S.K. (2008). Similarity measure of soft sets. New Math. Nat. Comput..

[18] Liu Z., Qin K., Pei Z. (2014). Similarity measure and entropy of fuzzy soft sets. Sci. World J..

[19] Jiang Y., Tang Y., Liu H., Chen Z. (2013). Entropy on intuitionistic fuzzy soft sets and on interval-valued fuzzy soft sets. Inf. Sci..

[20] Broumi S., Smarandache F. (2013). Several similarity measures of neutrosophic sets. Neutrosophic Sets Syst..

[21] Ye J. (2014). Similarity measures between interval neutrosophic sets and their applications in multicriteira decision-making. J. Intell. Fuzzy Syst..

[22] Zadeh L.A. (1968). Probability measure of fuzzy events. J. Math. Anal. Appl..

[23] De Luca A., Termini S. (1972). A definition of a nonprobabilistic entropy in the setting of fuzzy set theory. Inf. Control.

[24] Burillo P., Bustince H. (1996). Entropy on intuitionistic fuzzy sets and on interval-valued fuzzy sets. Fuzzy Sets Syst..

[25] Selvachandran G., Maji P.K., Faisal R.Q., Salleh A.R. (2017). Distance and distance induced intuitionistic entropy of generalized intuitionistic fuzzy soft sets. Appl. Intell..

[26] Majumdar P., Samanta S.K. (2014). On similarity and entropy of neutrosophic sets. J. Intell Fuzzy Syst..

[27] Şahin R., Küçük A. (2014). On similarity and entropy of neutrosophic soft sets. J. Intell Fuzzy Syst..

[28] Garg H., Nancy (2017). Some new biparametric distance measures on single-valued neutrosophic sets with applications to pattern recognition and medical diagnosis. Information.

[29] Aydoğdu A. (2015). On Similarity and entropy of single valued neutrosophic sets. Gen. Math. Notes.

[30] Ye J. (2014). Single valued neutrosophic cross-entropy for multicriteria decision making problems. Appl. Math. Model.

[31] Garg H., Nancy (2016). On single-valued neutrosophic entropy of order *α*. Neutrosophic Sets Syst..

[32] Selvachandran G., Garg H., Quek S.G. (2018). Vague entropy measure for complex vague soft sets. Entropy.

[33] Selvachandran G., Quek S.G., Smarandache F., Broumi S. (2018). An extended technique for order preference by similarity to an ideal solution (TOPSIS) with maximizing deviation method based on integrated weight measure for single-valued neutrosophic sets. Symmetry.

[34] Ye J., Du S. (2017). Some distances, similarity and entropy measures for interval-valued neutrosophic sets and their relationship. Int. J. Mach. Learn. Cyber..

[35] Pramanik S., Dey P.P., Smarandache F., Ye J. (2018). Cross entropy measure of bipolar and interval bipolar neutrosophic sets and their application for multi-attribute decision making. Axioms.

[36] Ye J., Cui W. (2018). Exponential entropy for simplified neutrosophic sets and its application in decision making. Entropy.

[37] Cui W., Ye J. (2018). Improved symmetry measures of simplified neutrosophic sets and their decision-making method based on a sine entropy weight model. Symmetry.

[38] Selvachandran G., Garg H., Alaroud M.H.S., Salleh A.R. (2018). Similarity measure of complex vague soft sets and its application to pattern recognition. Int. J. Fuzzy syst..

[39] Zhang Q., Xing H., Liu F., Ye J., Tang P. (2014). Some new entropy measures for interval-valued intuitionistic fuzzy sets based on distances and their relationships with similarity and inclusion measures. Inform. Sci..

[40] Tu A., Ye J., Wang B. (2018). Multiple attribute decision-making method using similarity measures of neutrosophic cubic sets. Symmetry.

[41] Cui W., Ye J. (2018). Multiple-attribute decision-making method using similarity measures of hesitant linguistic neutrosophic numbers regarding least common multiple cardinality. Symmetry.

[42] Smarandache F. (1999). A Unifying Field in Logics. Neutrosophy: Neutrosophic Probability, Set and Logic.

